# Embedding Research on Implementation of Primary Health Care Systems Strengthening: A Commentary on Collaborative Experiences in Ethiopia, Ghana, and Mozambique

**DOI:** 10.9745/GHSP-D-22-00061

**Published:** 2022-09-15

**Authors:** African Health Initiative Partnership Collaborative for Embedded Implementation Research

**Affiliations:** ^a^Members listed at the end of the article.

## Abstract

Achieving universal health care coverage requires the adoption of primary health care policies and delivery strategies that are evidence based. Although this has been confronted by manifold challenges, particularly in the health systems of sub-Saharan Africa, there are promising approaches for accomplishing this objective. Salient among these is embedding implementation research (i.e., the study of methods to promote the systematic uptake of evidence-based interventions (EBIs) into routine practice) into policy making and implementation processes. Since 2007, the African Health Initiative of the Doris Duke Charitable Foundation supported partnerships that strengthened primary health systems and policy implementation in 7 countries in sub-Saharan Africa using the embedded implementation research as a core strategy. This programmatic review and analysis aims to identify the core features and processes that characterized how the partnerships operationalized the embedded implementation research approach and understand the factors that helped and constrained partnerships’ effective use of this approach. For this, we drew upon findings from a desk review that consisted of 30 examples of embedded implementation research conducted by 3 African Health Initiative partnerships between 2016 and 2021 in Ethiopia, Ghana, and Mozambique. In addition, we conducted and analyzed 13 in-depth interviews with embedded implementation research stakeholders of the 3 projects. Core features and processes of embedded implementation research were: (1) the leadership role of policy decision makers and implementation leaders; (2) positioning research with program implementation at multiple levels of health systems; (3) multidisciplinary and multisectoral partnerships; (4) focus on research capacity building; and (5) real-time feedback loops and knowledge translation. Factors influencing the effectiveness of the embedded implementation research experiences involved: (1) the implementation climate and leadership; (2) opportunities and capacities to circulate and absorb new information; and (3) stakeholders’ baseline knowledge and embedded scientists’ identification within their organizations.

## Abstract (precis)

The African Health Initiative prioritized embedded implementation research using a multidisciplinary partnership model that empowered decision makers and embedded research and capacity building at multiple levels of health systems.

## Key Messages

- Engaging policy decision makers and implementation teams elicited critical input on root causes of implementation problems, prioritized which factors to address and how, and determined what questions to ask and answer to best guide primary health care policy implementation.
- Integrating decision makers in program research leadership roles allowed data collection, analysis, and dissemination to align with planning cycles during which new findings could be reflected on pragmatically and, if appropriate, used to guide program implementation.
- Multidisciplinary partnerships between policy decision makers, implementation leaders from district health systems, and local research institutes fostered collaboration in cocreating implementation strategies and research plans and emphasized knowledge and experience sharing.
- Embedded IR was influenced not just by individual leadership and local implementation climate but the overarching organizational culture in which the African Health Initiative partnerships were themselves embedded nationally.
- The degree to which embedded IR leads to meaningful and lasting change in health systems is affected by opportunities for, and impediments to, disseminating and absorbing new knowledge.

Resumo em português no final do artigo.

## BACKGROUND

To achieve universal health coverage (UHC) despite financial, human, and infrastructural resource constraints, sub-Saharan African country health systems must implement primary health care (PHC) policies and delivery strategies that are evidence based.^1^^–^^14^ Despite wide recognition of this, efforts to strengthen health systems based on evidence are beset by challenges. Currently, PHC policy and implementation research are limited, particularly relative to the scope and severity of primary health and systems challenges in sub-Saharan Africa.^15^^,^^16^ Most research output focusing on health care delivery and health systems comes from research institutions based in other continents. Accordingly, global calls have directed attention to the need to build research capacity and leadership in sub-Saharan Africa at the institutional and leadership levels.^17^^,^^18^ These initiatives have advanced a vision of situating research within health systems and communities in a manner that addresses structural inequities,^19^ maximizes the benefits of new knowledge for staff of local health systems, and enhances the role of “research consumers” in the design and use of research.^18^^,^^20^ Although this effort has achieved some success, strategies for translating evidence into large-scale practice in sub-Saharan Africa are often poorly suited to the complexities of local health systems, do not engage key stakeholders before and during the research process,^18^^,^^20^ and struggle due to lengthy time lags in applying research to design and accelerate scale-up of PHC reforms.^21^^–^^24^

Implementation research (IR)—defined as the study of methods to promote the systematic uptake of evidence-based interventions (EBIs) into routine practice—can be useful in achieving this goal because it adopts these processes as the subject of scientific inquiry.^25^ IR acknowledges the influence of culture, context, and nonlinear organizational tendencies on knowledge translation, and the need for implementation strategies to support EBI implementation that are iterative, ongoing, and adaptive to local environments.^26^ Therefore, understanding how to optimally use IR in sub-Saharan African country health systems is key to achieving UHC. One approach that has been of increasing interest is “embedded IR.”^27^ Embedded IR emphasizes IR capacity strengthening, partnerships, and co-design. It elevates the role of policy decision makers and implementation leaders to identify implementation challenges, prioritize evidence needs, and coproduce and colead work for dissemination and scale. In addition, IR seconds local scientists to help study implementation challenges from a position embedded within implementation processes in collaboration with implementers and decision makers and, with this, promotes the uptake of research findings into policy and implementation decision making.^28^

Embedded IR elevates the role of policy decision makers and implementation leaders to identify implementation challenges, prioritize evidence needs, and lead dissemination and scale.

## THE AFRICAN HEALTH INITIATIVE

Since 2009, the African Health Initiative (AHI) of the Doris Duke Charitable Foundation (DDCF) has supported collaborations between Ministries of Health (MOHs), PHC implementation leaders, and academic research groups to embed IR into their work to strengthen and learn from PHC systems in 6 countries.^29^ The first phase of the AHI (2009–2014) demonstrated the “proof of concept” that locally adapted PHC implementation strategies could effectively accelerate progress towards UHC in Ghana, Mozambique, Rwanda, Tanzania, and Zambia. The second phase, which started in 2015, focused on replication and supporting the scale-up of effective strategies in 3 countries—Ghana, Mozambique, and Ethiopia (whose country partnership joined the AHI at that time).^30^ Although similar in their core principles, AHI partnerships differed with respect to their specific implementation strategies and activities, which ranged from data quality improvement and use to strengthening health facility management to improving community-based PHC systems. However, embedded IR was a common strategy across the 3 AHI partnerships during Phase 2 (Box).^31^^–^^35^

BOX **Embedded Implementation Research Strategy Used by African Health Initiative Partnership in 3 Countries**

**Ethiopia Data Use Program (DUP):** In Ethiopia, the Ministry of Health (MOH) launched the Health Sector Transformation Plan (2015–2020), which called for an “information revolution,” particularly the digitization of health information systems (HIS) and building capacity to use HIS data and ensure data quality in primary health care settings. The Connected Woreda strategy of the MOH emerged as the national guide for achieving the plan’s vision of creating an information-use culture at all levels of the health care system.

To help implement that strategy, in 2017, the MOH—in partnership with John Snow Research and Training Institute, Inc., and its subpartners, Regenstrief Institute, University of Gondar, and Gobee Group—launched the DUP, with initial funding from the Bill & Melinda Gates Foundation, and, in 2018, additional funding from the Doris Duke Charitable Foundation. DUP has helped establish systems to strengthen the national HIS for supporting data quality and use and demonstrate how to effectively intervene to that effect in primary health care systems throughout the country. A core component of the DUP is an academic–implementer partnership model, called the Capacity Building and Mentoring Program, wherein researchers receive graduate training in implementation research (IR); as part of this training, they partner with regional health bureaus to collaborate on priority research questions to inform and/or strengthen service delivery. In partnership with the regional health bureaus under the leadership of the MOH, 6 local universities (Addis Ababa University, University of Gondar, Mekelle University, Jimma University, Hawassa University, and Haramaya University) played a key role in establishing the Capacity Building and Mentoring Program.

**Community-based Health Planning and Services+ (CHPS+):** In Ghana, the national program health care program, Community-based Health Planning and Services (CHPS) emerged from a series of studies in the 1990s–2000s that showed that combining the deployment of paid, multitasked community health officers with community mobilization could reduce child mortality. National policies scaling up CHPS comprised isolated activities that lacked “unifying systems thinking.”^31^ To accelerate the slow scale-up of CHPS and address this lack, during African Health Initiative Phase 1, the Ghana Essential Health Interventions Program was launched in the Upper East Region and designed and demonstrated the health impact of a package of interventions that strengthened district health systems and made it easier to put CHPS into practice and reduce child mortality.^32^ In Phase 2, collaborators replicated and scaled the model, now called CHPS+, which conducted a multicomponent strategy in districts with low CHPS coverage to help accelerate scale-up.^33^ Key pieces of the intervention at the district level involved training and mentorship to health care workers and community health workers, leadership capacity building, monitoring and evaluation systems strengthening, flexible catalytic funding, and district-to-district peer exchanges. At the national level, CHPS+ also intervened to help build knowledge management capacity and promote evidence use during policy decision making.

**Integrated District Evidence-to-Action program to improve maternal, newborn, and child health (IDEAs):** In Mozambique, the Phase 1 partnership supported the MOH’s decentralization of management of public health sector resources in 12 districts of Sofala province. This partnership focused on strengthening routine HIS, improving data quality and its use through appropriate tools to facilitate decision making by health system managers, strengthening management and planning capacity, funding district health plans, and building capacity for operations research.^34^^,^^35^ In 2017, the partnership replicated the program, called the IDEAs program, in the neighboring Manica province; this project builds on improvements in data quality and management capacity achieved in Phase 1 by introducing an enhanced audit and feedback strategy to improve how facilities in the 2 provinces implement the MOH’s essential package of interventions to reduce maternal, child, and newborn deaths. In addition, the Phase 2 partnership implemented a 2-prong approach to foster IR capabilities among Government of Mozambique institutions and personnel: (1) a suite of trainings on IR (including both degree-offering training at the doctoral and master’s levels, as well as short courses); and (2) multiple rounds of sourcing IR priorities from provincial leadership and supporting Mozambican public sector research institutions to develop, implement, and disseminate IR addressing these priorities.

We reflect on how AHI partners carried out embedded IR in Phase 2 of the AHI. In doing so, we describe the features and processes of embedding IR and the factors that impeded or enhanced these endeavors, and we give insights into how embedded IR can support UHC achievement in sub-Saharan African country health systems in the future.

To identify the core features and processes that AHI partnerships used to embed IR in PHC systems, we drew upon findings from a desk review of 30 examples of embedded IR. We applied framework analysis to the data, which involved sifting and sorting data—first, to familiarize ourselves with the data and identify salient concepts and relationships among them; then, to determine an appropriate thematic framework.^34^ For the second step, we benefited from the conceptual guidance of Varallyay et al., specifically a framework for evaluating the processes of embedded IR.^35^ Employing the constructs supplied by this framework, we then indexed textual data from desk review materials by coding segments of text into thematic categories. Next, we charted the segments of coded text, rearranging them into charts defined by the respective themes, and then we mapped and interpreted the key characteristics observed in the data as laid out in charts.

To help understand the barriers and facilitators of using the embedded IR approach, we conducted and analyzed 13 in-depth interviews with stakeholders of the 3 projects (3 policy makers, 7 embedded researchers, and 3 implementation team leaders). For this analysis, we also used frameworks analysis, employing the same steps as in the desk review; however, with these data, we drew upon the domains and constructs from the Consolidated Framework for Implementation Research to adapt a thematic framework.^36^

## CORE FEATURES AND PROCESSES OF EMBEDDED IR

The Table provides an overview of AHI Phase 2 projects and the way that each employed embedded IR in large-scale PHC programs to assess whether implementation strategies effectively improved EBI implementation. All projects embedded IR in PHC systems to enhance public sector adoption and implementation of national policy frameworks and intervention packages. Most studies investigated the barriers and facilitators to implementation effectiveness and/or described implementation practices that were effective (n=20); 6 of these studies were conducted to inform the adaptation of implementation strategies. Fewer examples of embedded IR evaluated whether strategies were associated with change or differences in implementation outcomes (n=4). Collectively, the 3 partnerships illuminate important lessons on the core features and processes of embedded IR, which is effective in achieving the IR goals.

All projects embedded IR in PHC systems to enhance public sector adoption and implementation of national policy frameworks and intervention packages.

**TABLE.** Use of Embedded IR During African Health Initiative Phase 2 in Ethiopia, Ghana, and Mozambique

	**Ethiopia (Data Use Project)**	**Ghana (CHPS+)**	**Mozambique (IDEAs)**
**Target program**	Health Sector Transformation Plan 1 (2015–2020), which called for an Information Revolution to create an information-use culture at all levels of the health care system.	The national PHC program, CHPS.	The national maternal, newborn, and child health programs, led by the MOH.
**Evidence-based intervention**	Data quality improvement and use for point-of-care decision making in PHC.	CHPS+, the health interventions package (Ghana Essential Health Interventions Program) that strengthened CHPS in Phase 1.	MOH-adopted essential interventions to reduce maternal, child, and newborn deaths.
**Implementation strategy**	The Data Use Project has supported several interventions, including the Capacity Building and Mentorship Program. The key focus areas were promoting use of quality data for decision making, digitization, and strengthening governance of HIS and implementation research for evidence-based programming.	CHPS+ comprises a combination of approaches, including peer exchanges between “systems learning districts” and “trainee districts” to transfer best practices for putting lessons from the Ghana Essential Health Interventions Program into place and flexible catalytic funding for implementation teams in systems learning and trainee districts to make implementation changes based on lessons learned.	Building on data quality and use improvements in Phase 1 and the MOH prioritization of maternal, child, and newborn interventions, IDEAs implemented an enhanced A&F strategy to link district and facility management teams and promote facility teams’ use of data to plan, set goals, track, and benchmark progress vis-à-vis implementation of adopted interventions to reduce maternal, child, and newborn mortality.
**Health system level(s)**	At national and regional levels, covering all regions of Ethiopia. Data were also collected from selected sites in Addis Ababa City administration to generate qualitative data on point-of-care data use practices within PHC.	In Northern and Volta regions of Ghana, 4 “systems learning” and trainee districts. IR engages district health management teams, CHPS supervisors at district and subdistrict levels, community health officers, community health management committees, and volunteers.	In 7 districts in Manica and Sofala provinces IR engages national, provincial, district, and facility health system leadership and PHC workers at facilities.
**Examples of research question(s)**	Do interventions designed and implemented under the Information Revolution improve HIS performance? What are the factors that help or hinder the effectiveness of Information Revolution interventions at the point of care?	What systems weaknesses, implementation challenges, and capacity gaps should be prioritized to best support the spread of CHPS+ in “trainee districts”? Were CHPS+ interventions acceptable and feasible? Do stakeholders perceive them as effective? Do these interventions contribute to measurable changes in health services and population outcomes?	What factors explain poor maternal and newborn outcomes and care practices? What factors help or hinder the IDEAs strategy to improve MNCH guideline implementation? Has MNCH guideline implementation improved during the project?
**Research team affiliation – policy decision maker**	Co-investigators were from the MOH Policy, Planning, Monitoring and Evaluation Division; Health Information Technology Directorates; and regional health bureaus.	Co-principal investigator of CHPS+ was the director of the Policy Planning, Monitoring and Evaluation Division of the Ghana Health Service.	Co-principal investigator is the national director for Public Health and at the MOH.
**Research team affiliation – implementation leader**	Co-investigator from John Snow Research and Training Institute, Inc., Ethiopia Office oversaw research implementation. Staff of Regional Health Bureau participate in study design and interpretation of findings.	CHPS+ consults district-, facility-, and community-level PHC staff multiple times per year to obtain insight on implementation challenges and prioritize those that require deeper investigation.	IR priorities are elicited annually from provincial leadership nationwide. National research institutions were engaged to codevelop protocols with provincial health authorities, as well as conduct and disseminate research as co-investigators on these studies. National, provincial, and district leaders were selected for degree-offering training in public health and implementation science at national universities and at the University of Washington.
**Research team affiliation – research partner**	Co-investigators from Addis Ababa University, University of Gondar, Mekelle University, Hawassa University, Haramaya University, and Jimma University.	Co-principal investigator of CHPS+ from University of Ghana, University of Development Studies, and University of Health and Allied Sciences train PHC staff in CHPS+ districts on IR.	Co-principal investigators from national research institutions, including the National Institute of Health, Beira Operations Research Center, and provincial research nuclei. The national research institutions trained district and provincial health staff in embedded IR on MNCH guideline implementation.
**Methods**	Qualitative semistructured interviews and focus group discussions; analysis of HIS data.	Semistructured interviews and focus group discussion conducted every year. Baseline, midline, and endline household survey data used to gauge community perceptions of CHPS.	Qualitative semistructured interviews and focus group discussions, analysis of quantitative data from HIS, supervision reports, and facility assessments.
**Examples of findings**	Qualitative data suggest that data quality and use improved due to IR implementation. Drivers of this were the way in which performance management teams engaged leaders, coaching, and mentoring of PHC workers at the point of care and use of incentives. Barriers to optimal data use practices and quality were the presence of multiple and sometimes duplicative HIS tools in facilities, weak HIS infrastructure, paucity of HIS technicians, and negative attitudes about data among PHC workers.	Baseline qualitative and survey research identified PHC worker shortages, poor community engagement practices, lapses in supply chain, and lack of essential transportation. Capacity development priorities were logistics management, community engagement, and management of referrals at the CHPS level. Midline qualitative assessments indicated that CHPS+ was acceptable and effective at promoting learning, cooperation, planning, and adapting good practices—importantly, practices related to district stakeholder engagement for resource mobilization. This helped trainee districts to address capacity gaps, fill some resource gaps, and improve community engagement.	Factors that helped the IDEAs A&F strategy were a positive and encouraging learning environment, strong networks for communication and circulation of new information, and strong leadership of district managers and supervisors especially with respect to applying pressure and motivating performance improvement at facilities. Barriers were the inability of the intervention to address financial and human resource constraints that undermine performance improvement, as well as implementers’ perception that the intervention was narrowly focused on MNCH guidelines and should instead address a wider range of PHC challenges.
**Policy/program recommendations**	To establish an information use culture in the health system, the MOH should invest in strategies to build capacity of health care workers in data visualization and interpretation, expand the presence of performance monitoring teams, and use a combination of monetary and nonmonetary incentives to promote data use at the point of care.	Combining peer exchanges with catalytic funding supports adaptation and scale-up of CHPS; however, structural interventions remain necessary to address chronic workforce and logistical problems that undermine the sustainability of improvements achieved and constrain impact.	Leaders should establish an enabling environment for performance improvement by promoting opportunities to learn, adapt, and explore new ways to solve problems; supporting information and experience sharing; and making visible efforts to motivate implementation teams and hold them accountable for performance. To be most effective, A&F strategies should address both national and local priorities.

Abbreviations: A&F, audit and feedback; CHPS, Community-based Health Planning and Services Initiative; HIS, health information system; IDEAs, Integrated District Evidence-to-Action program to improve maternal, newborn, and child health; IR, implementation research; MNCH, maternal, newborn, and child health; MOH, Ministry of Health; PHC, primary health care.

### Role of Policy Decision Makers and Implementation Teams in Research

In all the projects, research partners engaged decision makers from policy and implementation settings in deliberating on findings from formative research or needs assessments. During this engagement, decision makers provided critical input on root causes of implementation problems, prioritized which factors to address and how, and determined what questions to ask and answer to best guide PHC policy implementation. As such, decision makers were both coproducers and consumers of embedded IR. During implementation, researchers engaged decision makers in interpreting findings from embedded IR, which helped guide adaptations in implementation strategy, identification of important implementation issues and topics for new embedded IR, and issuance of recommendations for policy improvements. In Mozambique, the Integrated District Evidence-to-Action program to improve maternal, newborn, and child health (IDEAs) research team, which is led by the principal investigator who is also the National Director of Public Health at the MOH, conducted semiannual meetings with district and facility teams in which they shared and collectively reflected on action plan achievements. These events provided opportunities for stakeholders to identify relevant implementation issues and research questions.^37^

### Positioning Research Within Program Implementation at Multiple Levels

Placing decision makers in leadership roles in research projects enabled partners to integrate the research and program processes. This integration allowed their data collection, analysis, and dissemination to align with planning cycles during which new findings could be reflected on pragmatically and, if appropriate, used to guide PHC program implementation. In Ethiopia, the DUP worked as an integrated project within the MOH and regional health bureaus to develop the enabling policy “road map” by developing a national eHealth architecture, convening platforms to create alignment among health information system (HIS) actors, and supporting systems to facilitate data exchanges and planning implementation. One example is the embedded IR study on how to optimize electronic community HIS and support its implementation and scale-up. This study was codesigned and overseen by MOH staff from the Policy, Planning, Monitoring and Evaluation Division and Health Information Technology Directorate, who timed the work so that results would be available for decision makers to consider as they plan to scale up the electronic community HIS.^38^

### Multidisciplinary and Multisectoral Partnerships

AHI partnerships fostered collaboration between policy decision makers, implementation leaders from district health systems, local research institutes, and, in some instances, communities. Cooperation among these actors started with using preliminary data on the EBI context to cocreate implementation strategies and coproducing a research agenda that met the information needs of decision makers from different levels of the health system. In Ghana, researchers from the University of Development Studies (UDS) and University of Health and Allied Sciences (UHAS) elicited the perspectives of community members and community-based PHC workers on problems underlying suboptimal Community-based Health Planning and Services (CHPS) implementation, particularly in terms of community entry and engagement practices. These actors cocreated a strategic response to catalyze better adoption and spread of CHPS in trainee districts, which was then monitored and evaluated during Phase 2.^39^^–^^41^ The implementation of CHPS+ is led by the Ghana Health Service Policy, Planning, Monitoring and Evaluation Division, which uses embedded IR results during deliberations on strategy to strengthen implementation and further scale-up of CHPS countrywide.

### Focus on Research Capacity Building

The partnerships incorporated research capacity building into embedded IR cycles. In Ethiopia, local universities offer pre-service and in-service trainings for health workers and managers that work in PHC settings to build their capacity to manage and use health information and enhance HIS staff opportunities, as well as eLearning courses on developing and conducting small-scale IR projects. In Ghana, junior researchers were paired with more experienced research staff during data collection and analysis. UDS and UHAS staff led IR trainings with members of district health teams. Additionally, UDS and UHAS coordinated embedded IR as part of their applied implementation science training. The Mozambique partnership project channels seed funding to national research institutions to codesign, implement, and disseminate IR protocols that respond to provincial research priorities identified through annual solicitations from the MOH. All partnerships dedicate resources to support the training of numerous junior investigators to receive Master’s degrees (57 in Ethiopia, 36 in Ghana, and 10 in Mozambique) and PhDs (8 in Ethiopia, 7 in Ghana, and 5 in Mozambique), mostly at national universities.

### Real-Time Feedback Loops and Knowledge Translation

The implementation processes used by each partnership emphasize knowledge and experience sharing. Data from embedded IR are circulated among partners and research expertise is embedded within implementation and decision-making cycles. These practices help promote the timeliness of data interpretation, as well as opportunities to translate IR findings into programmatic alternatives that reflect decision makers’ priorities and concerns and are actionable through routine systems. The IDEAs implementation process exemplifies this. This project’s evaluation focuses on whether a district-led audit and feedback strategy improves the implementation of essential maternal, child, and newborn health interventions. Embedded researchers from the IDEAs team give routine support to facility teams on managing routine data, ensuring data quality, measuring progress, and visualizing results. They also share findings from qualitative assessments of implementation with facility teams and district managers to guide how they address barriers and facilitators to effective implementation. District authorities convene meetings quarterly, during which facility managers present their progress and deliberate on the root causes of challenges and what to do about them; also at these meetings, local decision makers ascertain whether implementation of the essential interventions has improved and give guidance on next steps. Teams develop new action plans, accordingly, and embedded researchers conduct qualitative investigations on the factors that influence whether these activities improve services.^37^

The implementation processes used by each partnership emphasize knowledge and experience sharing.

## BARRIERS AND FACILITATORS OF EMBEDDED IR

In-depth interviews identified contextual factors that influenced the effectiveness of AHI partnerships’ embedded IR.

### Implementation Climate and Leadership

The implementation climate, depending on how leaders manage it, either enhanced or created obstacles for embedded IR. Stakeholders from all partnerships were able to provide examples of both.

*The thing that distinguishes success from failure or from- you know- the business-as-usual type of work, is very strong leaders who are able to work within a system that is governed by tradition, and norms but challenge those norms and traditions in ways that can be used to improve the system. That’s critical.* —Policy maker, Ghana

The context of embedded IR was often defined by implementation leaders’ experience and skills at balancing adherence to procedures of national health care bureaucracies, on the one hand, with adaptiveness to new lessons and openness to change, on the other. By cultivating an implementation climate that promotes timely sharing of data and information, trying new approaches, and circulating goals and feedback, leaders help establish a healthy tension for change. The benefits of these efforts are clear in Mozambique.

*At national level [there is] a platform [for stakeholders and MOH] to discuss the main findings of [embedded IR] and disseminate the best practices. It’s part of improving the health system in a way that can improve the way of delivering health to the people.* —Policy maker, Mozambique

By cultivating an implementation climate that promotes timely sharing of data and information, trying new approaches, and circulating goals and feedback, leaders help establish a healthy tension for change.

In the 3 countries, embedded IR was influenced not just by individual leadership and local implementation climate but also by the overarching organizational culture in which the AHI partnerships were themselves embedded nationally. In Ethiopia, the MOH declared an “Information Revolution,” which as an implementation team leader stated, was aimed at “bringing fundamental cultural and attitudinal change regarding the practice of collecting data and how it is used.” And blending these changes with the introduction or adaptation of “tools for analyzing data and using it for decision making at [the service delivery] level.”

Visible, high-level commitment to this component of the Health Sector Transformation Plan instigated helpful normative change about data and research utilization that enhanced motivation to practice embedded IR throughout the health sector. However, stakeholders reported difficulty in managing collaborative research partnerships when embedded IR members from the government do not remain in their positions for a long time.

*One disadvantage that earlier is since we started this project, the leadership at the Ministry of Health level changes very often. We’re (now) dealing with the third person. So that’s had an implication on the performance of the whole embedded research [project]. So having consistent leadership within the ministry is also critical.* —Researcher, Ethiopia

### Opportunities and Capacities to Circulate and Absorb New Information

The degree to which embedded IR leads to meaningful and lasting change in health systems is affected by opportunities for, and impediments to, disseminating and absorbing new knowledge. In Ghana, implementation teams from trainee districts—that had embedded research in the formative context of adapting CHPS—drew additional benefit from exchanges with peers from systems learning districts.

*From the peer exchange [the implementation teams] learned from the successes of peers on how they overcome some of the same challenges … After coming to the peer exchange, they [the implementation team] came back and did a lot, they ensured that their entire district was able to go out and mobilize their communities—not just for them to provide accommodation and other things—but also to provide the services themselves.* — Implementation leader, Ghana

In Mozambique, district leaders combined facility-team exchanges, joint action planning, and public benchmark reporting to motivate quality improvement and adoption of priority interventions to reduce maternal, child, and newborn deaths. In that instance, implementation teams also benefited from associating with peers with whom their interactions are otherwise limited.

*Performance review helps very much to see if we are working or not, and presentations help to share experiences. If others did were successful, they show us the data … we can learn from them … This [interaction] helps develop action plans to address shortcomings. The plans are posted at a visible location in the health facility.* —Implementation team leader, Mozambique

All projects lamented the tendency for decision makers to be overwhelmed with multiple responsibilities, which slowed the pace of evidence sharing within their circles and undermined their collective capacity to make evidence-informed decisions.

*Like most of the time, those people from the ministry or like decision makers are implementing this. They are mostly busy people. They have so many things on the table: in addition to leading all the work on the field for those decision makers, there are so many meetings, they have so many management activities. So, the main challenge is their lack of time for the research project. I think that is the major obstacle.* —Researcher, Ethiopia

### Stakeholders’ Baseline Knowledge/Capacities and Embedded Scientist Identification Within Organization(s)

Embedded researchers reported difficulties im-parting adequate knowledge on the principles of IR and an appreciation for how it might strengthen policy making and implementation.

*This will be solved, but during our initial engagement, that became a critical challenge because most of these policy makers, they have a very limited exposure to research, so they fail to understand the importance of focus and the importance of being very specific.* —Researcher, Ethiopia

Positioning research within programs implies the necessity for policy makers and implementers to embrace the scientific process and blend clinical and bureaucratic processes with creative inquiry. Doing so requires acquisition of new skills and the ability to adapt customary ways of working. It follows that uptake of embedded IR was in some instances impeded by passive resistance to the shift away from “business as usual.”

*You know that when you start, methodology is required to shift the way that you are doing things. This is a challenge in a way that people will move from what is usual to another way of doing things. It’s required to have to train people on how to draw the protocols but also about implementing.* —Researcher, Mozambique

Uptake of embedded IR was in some instances impeded by passive resistance to the shift away from “business as usual.”

In addition, embedded researchers described their struggle in negotiating their standing and sense of incorporation within academia and health systems. Embedding research that is context-driven and maintaining its position within programs demands that researchers conform to the procedures and culture of adopting organizations. Some participants described that doing so conflicts with the goal of being impartial and producing objective, generalizable evidence.

*One critical challenge is to be able to tow that fine line between a researcher and then the implementer so that while you are trying to provide an objective assessment of what is going on, you don't slip on to the other side where you sort of compromise the independence. The researcher is … torn between the issue of complete independence and the fact that you are a partner in this process.* —Researcher, Ghana

## LESSONS LEARNED

In synthesizing the embedded IR experiences across the 3 AHI Phase 2 partnerships, we noted lessons on 5 key themes.

### Role of Decision Makers

The intellectual stewardship and direction carried out by decision makers are crucial to the success of research in improving programs; however, it is important to engage leaders of policy decision making (usually at the national level) and policy implementation (usually at the local level) to maximize the relevance of IR and ensure that the research is integrated within decision-making and implementation processes. For this effort to succeed, decision makers should be engaged early and often, and academic partnerships are required to strengthen research capacity at both levels. When possible, researchers should engage multiple decision makers and contribute to building research capacity in groups so that embedded IR is not destabilized when staff turnover occurs. Health sector funders should help establish permanent and active units within the MOH that manage a PHC learning agenda, including partnerships with academic groups and implementation partners; coordinate research capacity building activities; and integrate embedded IR policy- and decision-making processes. Donors should target these structures with solicitations for embedded IR proposals.

### Positioning Research Within Program Processes

Leaders from national and local levels should consider the timing of planning and decision-making processes that should be informed by embedded IR to maximize the prospect that evidence is available when it is most needed. In addition, decision makers and scientists should prioritize activities that expand opportunities and strengthen capacities to circulate and absorb evidence from local embedded IR or relevant evidence from similar settings. Funders should require that MOH and partners integrate IR into health sector strategic plan-funding applications and ask that proposals incorporate local evidence. Similarly, health sector funders and MOH should encourage that planning frameworks call on districts to propose embedded IR in annual plans.

Decision makers and scientists should prioritize activities that expand opportunities and strengthen capacities to circulate and absorb evidence from local embedded IR or relevant evidence from similar settings.

### Collaborative, Multidisciplinary Research Partnerships

Embedded IR requires that research teams have an appropriate skill mix to carry out high-quality science, adapt methods so that they are contextually appropriate and fit for purpose, and ensure that data collection and use are integrated within processes of routine implementation. This requires partnerships that bring together scholars, implementers, and policy makers. Achieving optimal collaborative arrangements and dynamics takes time, and funders should help nurture these relationships by promoting opportunities for decision makers and implementation leaders to learn about IR and to interact informally with researchers—generating spontaneous opportunities for advancing partnerships. Clarity on roles and expectations is critical to achieve at the outset. This includes openness about tensions that arise within embedded IR teams between “business as usual” preferences and the imperative to learn and adapt, as well as a willingness to expect and respond to systems failures when they occur.

### Research Capacity Building

Ensuring that actors from the countries that host embedded IR can independently conduct high-quality research is essential for parlaying embedded IR into long-term progress toward UHC. Embedded IR teams from sub-Saharan African countries should articulate the support that they require to do such research and the existing institutions to invest in; they should also have leeway in adapting research capacity-building programs to reflect local context and capacity. Donors should be responsive to these appeals and consider funding strategies that demonstrate how research capacity building strengthens health systems. Research capacity building should incorporate a continuum of activities including designing protocols and analysis methods, facilitating knowledge translation, and publishing in journals. Training opportunities should be situated in health program implementation to further strengthen essential partnerships. Whereas strengthening capacity of scientists in research institutions is critical, so too is the imperative to adapt training approaches to meet the needs of implementation leaders and decision makers, whose roles are equally important.

### Reporting Standards and Practices

Our investigation highlighted tensions experienced by researchers that seek to generate objective knowledge on how to strengthen health systems and are yet embedded as members of the implementation systems that they study from within. Experiences of researchers of other social sciences emphasize similar challenges in which researchers must contend with their positionality.^42^ Whereas guidance on conducting qualitative research delineates steps scientists should use to explore and explain their positionality to bolster the credibility and enhance interpretation of evidence, it appears that either IR is lacking in this regard or that such guidance is not well known or widely accessible to implementation researchers.^43^^,^^44^ Leaders of the embedded IR enterprise should consider ways to adapt reporting standards and guidelines to promote practices that help researchers address positionality and ensure research quality.

## CONCLUSION

All 3 AHI projects reported ways that embedded IR informs program implementation improvements and lauded the DDCF for prioritizing embedded IR as a core component of larger investments in accelerating the achievement of UHC. This support for embedded IR was at an adequate level with sufficient time frames and flexibility so that each project could conceptualize and embed IR, learn, and translate lessons into program action.^18^ DDCF support leveraged pre-existing, strong, multidisciplinary collaborations with IR experience. As a result, many of the common challenges that beset embedded IR were not identified in this review. However, some common challenges persisted, notably difficulties managing the implementation climate and expanding the absorptive capacity of bureaucracies to learn and adapt to new information.

As embedded IR partnerships mature, including relationships with funders, new solutions will be required to address these challenges. This should include a deeper commitment to research capacity building, flexible support for local partnerships to engage actors and use evidence to solve problems, and inventive strategies to make these opportunities more available for lesser costs. In the meantime, projects with funding for embedded IR can look to AHI for inspiration on how to maximize the impact of embedded IR using a multidisciplinary partnership model that empowers decision makers and embeds research and capacity building at multiple levels of health systems.

**AHI Partnership Collaborative for Embedded Implementation Research Authors:** Colin Baynes (Department of Global Health, University of Washington, Seattle, WA, USA); Mallory Sheff (Mailman School of Public Health, Columbia University, New York, USA); Lisa Hirschhorn (Feinberg School of Medicine, Northwestern University, Chicago, IL, USA); Lola Adedokun (Doris Duke Charitable Foundation, New York, USA); Quinhas Fernandes (Department of Global Health, University of Washington, Seattle, WA, USA; Ministry of Health, Maputo, Mozambique); Kenneth Sherr (Department of Global Health, University of Washington, Seattle, WA, USA); Sarah Gimbel (Department of Global Health, University of Washington, Seattle, WA, USA; Department of Child, Family, and Population Health Nursing, University of Washington, Seattle, WA, USA); Isaías Ramiro (Health Alliance International, Maputo, Mozambique); Sergio Chicumbe (Instituto Nacional de Saúde, Maputo, Mozambique); Eusebio Chaquisse (National Directorate of Public Health, Ministry of Health, Maputo, Mozambique); Nelia Manaca (Health Alliance International, Maputo, Mozambique); Orvalho Augusto (Department of Global Health, University of Washington, Seattle, WA, USA; Eduardo Mondlane University, Maputo, Mozambique); Hiwot Belay (Ethiopia Data Use Partnership, John Snow International, Addis Ababa, Ethiopia); Hibret Tilahun (Ethiopia Data Use Partnership, John Snow International, Addis Ababa, Ethiopia); Abebaw Gebeyehu (Ethiopia Data Use Partnership, John Snow International, Addis Ababa, Ethiopia); Afrah Mohammedsani (Ethiopia Data Use Partnership, John Snow International, Addis Ababa, Ethiopia); J. Koku Awoonor-Williams (Ghana Health Service, Accra, Ghana); Ayaga Bawah (University of Ghana, Accra, Ghana); James Phillips (Mailman School of Public Health, Columbia University, New York, USA); S. Patrick Kachur (Mailman School of Public Health, Columbia University, New York, USA); Pearl E. Kyei (University of Ghana, Accra, Ghana); Nicholas Kanlisi (Mailman School of Public Health, Columbia University, New York, USA); and Robert Alirigia (Ghana Health Service, Accra, Ghana).

## Translation

**Em Português**

**Incorporando a Pesquisa na Implementação do Fortalecimento dos Sistemas de Cuidados de Saúde Primários: Um Comentário sobre Experiências Colaborativas na Etiópia, Gana e Moçambique**

**Mensagens Chave**

• Nossa análise identificou lições aprendidas sobre os principais recursos e processos da pesquisa de implementação incorporada, incluindo a importância de: (1) o papel da liderança dos decisores de políticas e líderes de implementação na pesquisa de implementação; (2) posicionar a pesquisa com implementação de programas em vários níveis dos sistemas de saúde; (3) parcerias multidisciplinares e multissetoriais; (4) foco na capacitação em pesquisa; e (5) ciclos de feedback em tempo real e tradução do conhecimento.

• Os fatores que influenciam a eficácia das experiências da incorporação da pesquisa de implementação incluídas nesta revisão envolveram: (1) o clima de implementação e liderança; (2) oportunidades e capacidades para circular e absorver novas informações; e (3) conhecimento de base das partes interessadas e a identificação de cientistas integrados nas suas organizações.

**Resumo**

Alcançar a cobertura universal de saúde requer a adoção de políticas de cuidados de saúde primários e estratégias de prestação de serviços baseadas em evidências. Embora isso tenha sido confrontado por múltiplos desafios, particularmente nos sistemas de saúde da África sub-Sahariana, existem abordagens promissoras para atingir esse objetivo. Entre as abordagens, destaca-se a incorporação da pesquisa de implementação, ou seja, o estudo dos métodos para promover a adoção sistemática de intervenções baseadas em evidências (EBIs) na prática rotineira, na formulação de políticas e nos processos de implementação. Desde 2007, a Iniciativa Africana de Saúde da Fundação de Caridade da Doris Duke apoiou parcerias que fortaleceram os sistemas de saúde primários e a implementação de políticas em 7 países da África sub-Sahariana usando a pesquisa de implementação integrada como estratégia central. O propósito desta revisão e análise programática é de identificar os principais aspectos e processos que caracterizaram como as parcerias operacionalizaram a abordagem integrada da pesquisa de implementação e entender os factores que ajudaram e restringiram o uso efetivo das parcerias dessa abordagem. Para isso, baseamo-nos nas conclusões de uma revisão documental que consistiu em 30 exemplos de incorporação de pesquisa de implementação realizada por três parcerias da Iniciativa Africana de Saúde entre 2016-2021 na Etiópia, Gana e Moçambique. Além disso, realizamos e analisamos 13 entrevistas em profundidade com as partes interessadas na incorporação da pesquisa de implementação nos 3 projetos. As principais características e processos da pesquisa de implementação incorporada foram: (1) o papel da liderança dos decisores de políticas e líderes de implementação; (2) posicionar a pesquisa com a implementação dos programas em vários níveis dos sistemas de saúde; (3) parcerias multidisciplinares e multissetoriais; (4) foco na capacitação em pesquisa; e (5) ciclos de feedback em tempo real e tradução de conhecimento. Os fatores que influenciam a eficácia das experiências da incorporação da pesquisa de implementaçã envolveram: (1) o clima de implementação e liderança; (2) oportunidades e capacidades para circular e absorver novas informações; e (3) conhecimento de base das partes interessadas e a identificação de cientistas integrados nas suas organizações.

**Peer Reviewed**
